# Real-world COVID-19 vaccine effectiveness in Zimbabwe: A test-negative case-control study

**DOI:** 10.4102/jphia.v16i1.695

**Published:** 2025-02-19

**Authors:** Clara Haruzivishe, Nicholas Midzi, Tafadzwa Matanhire, Senga Sembuche, Masceline J. Mutsaka-Makuvaza, Rodgers R. Ayebare, Suzan Nakasendwa, Leah Mbabazi, Tonny Muwonge, Carl Mateta, Cynthia N. Chaibva, Calletta Gwatiringa, Kudzaishe E. Mutsaka Haruzivishe, Isaac Phiri, Tamrat Shaweno, Nebiyu Dereje, Tajudeen Raji, Mosoka P. Fallah, Munyaradzi Dobbie

**Affiliations:** 1 Department of Nursing Science, Faculty of Medicine and Health Sciences, University of Zimbabwe, Harare, Zimbabwe; 2 Africa Forum for Research and Education in Health (AFREhealth), Harare, Zimbabwe; 3 National Institute of Health Research, Ministry of Health and Childcare, Harare, Zimbabwe; 4 Research Support Center, University of Zimbabwe, Harare, Zimbabwe; 5 Africa Centers for Disease Control and Prevention, Addis Ababa, Ethiopia; 6 National Institute of Health Research, Ministry of Health Zimbabwe, Harare, Zimbabwe; 7 Infectious Diseases Institute, Makerere University, Kampala, Uganda; 8 Department of Epidemiology and Disease Control, Ministry of Health and Child Care, Harare, Zimbabwe; 9 Public Health Division, Ministry of Health and Child Care, Harare, Zimbabwe

**Keywords:** COVID 19, vaccine effectiveness, vaccination status, efficacy, co-morbidity health outcomes, hospitalisation

## Abstract

**Background:**

COVID-19 vaccination is critical in sub-Saharan Africa to reduce the disease burden. This study assessed real-world vaccine effectiveness (VE) in Zimbabwe.

**Aim:**

To determine COVID-19 VE and factors associated with disease severity and mortality in Zimbabwe.

**Setting:**

The study setting comprised a test-negative case-control study across health facilities in Harare and Bulawayo (May 2023 – August 2023).

**Methods:**

Adults (≥ 18 years) were recruited from COVID-19 registers (1:1 case-control; matched by sex, age and clinic visit date). Telephone interviews assessed vaccination status, disease severity (cases) and comorbidities. Conditional logistic regression estimated VE (1 – odds ratio*100), with stratification by age and comorbidities. Ordinal and simple logistic regression analysed factors associated with disease severity and vaccination–variant relationships.

**Results:**

Overall vaccination coverage was 38% (fully vaccinated including boosters), with 62% unvaccinated. The median age was 38 years (interquartile range [IQR]: 30–50) with more females (*n* = 352). Overall VE against any COVID-19 infection was 32.2% (95% CI: 8.9, 49.5). Older age (45+ years) and chronic conditions were associated with increased hospitalisation risk. Among cases, hospitalisation rate was 34.8% (*n* = 174/500) and COVID-19-related mortality rate was 11.6% (*n* = 58/500).

**Conclusion:**

This study found a moderate VE of COVID-19 vaccines in Zimbabwe, potentially influenced by age, comorbidities and variants. We highlight the need for targeted vaccination strategies and public health measures informed by these findings.

**Contribution:**

This research informs public health strategies to optimise vaccination efforts and improve health outcomes across Africa, aligning with the journal’s focus on public health issues.

## Introduction

The coronavirus disease 2019 (COVID-19) pandemic necessitated rapid development and deployment of vaccines to mitigate its spread.^1,2^ COVID-19 vaccines effectively reduce transmission, morbidity and mortality.^3^ Mass vaccination is a proven strategy for protecting susceptible individuals in the population.^4^ According to the World Health Organization (WHO) global vaccination target, Africa needed to vaccinate 70% of its population by 2022.^5^ However, by 2023 only 19% of the African population was fully vaccinated.^2^

The deployment of multiple COVID-19 vaccines, such as Sinopharm, Sinovac, Pfizer-BioNTech, Moderna, AstraZeneca, Johnson & Johnson and others, raised hope for mitigating the pandemic.^3^ Nonetheless, the primary objective of COVID-19 vaccination is not only to protect individuals from severe disease but also to reduce virus transmission.^4^ Numerous studies have also shown the importance of vaccination and the potential for herd immunity, which arises when a sufficient proportion of the population is immune, either through vaccination or previous infection.^5,6,7^ This threshold inhibits virus spread and ultimately safeguards those who cannot be vaccinated.^5^ As such, investigating vaccine effectiveness (VE) can also help understand whether a population can achieve herd immunity and determine whether more vaccination efforts are needed.^8,9^

Severe acute respiratory syndrome coronavirus 2 (SARS-CoV-2) has given rise to multiple variants of concern, including Delta and Omicron, which have raised questions about their impact on VE.^10,11^ Apart from mutations, VE is influenced by various factors, including population demographics, comorbidities, vaccination coverage, timing of vaccination and vaccine types.^3,12,13^ As such, understanding the factors associated with COVID-19 severity and mortality is of paramount importance in sub-Saharan Africa.^14^ In addition, individuals with underlying health conditions, such as diabetes, high blood pressure (HBP) and human immunodeficiency viruses (HIV), are at a higher risk of experiencing severe COVID-19.^15^ The prevalence of these comorbidities in sub-Saharan Africa influences disease outcomes. Moreover, the effectiveness of vaccines in reducing disease severity and mortality is a crucial consideration. The COVID-19 pandemic has underscored the importance of mass vaccination as a fundamental public health strategy. In Zimbabwe, the vaccination programme (Sinopharm and Sinovac vaccines were administered) was rolled out in February 2021, targeting at least 60% of the eligible population by December 2021.^16^ Thus, the study aimed to determine COVID-19 VE in Zimbabwe as well as determine the factors associated with COVID-19 disease severity.

## Research methods and design

### Study design

The study design was a test-negative retrospective case-control study comprising a historical cohort of community-dwelling individuals eligible for COVID-19 vaccination who presented to a testing/treatment centre meeting the local criteria for a patient under investigation for COVID-19 infection. Cases were adults (≥ 18 years) with laboratory-confirmed COVID-19 who presented to healthcare facilities with symptoms. Controls were adults who presented for COVID-19 testing but tested negative.

### Study setting

The study was conducted at central healthcare facilities in Harare and Bulawayo, Zimbabwe, from May 2023 to August 2023.

### Study population and sampling strategy

The study enrolled, from hospital records, 1000 participants aged 18 years and above with an available phone contact who had presented to the health care worker with at least one of the following symptoms: fever, cough, shortness of breath, and any other symptom based on the COVID-19 variant and country-specific criteria for suspected disease. In addition, all COVID-19 cases had a laboratory-confirmed diagnosis by polymerase chain reaction (PCR) or point-of-care test. Controls were participants presented at a facility to get a COVID-19 test but confirmed negative by PCR or point-of-care testing.

The study excluded individuals who had received a COVID-19 vaccine within 14 days of presentation to the testing and treatment centre. We classified participants as: (1) *vaccinated*: received two doses of a two-dose vaccine or one dose of a single-dose vaccine at least 14 days before presenting for a test; (2) *partially vaccinated*: received only one dose of a single-dose vaccine at least 14 days before they presented for a test; (3) *unvaccinated*: number of individuals who had not received a single dose of any of the available vaccines at the time of testing.

### Data-collection methods

#### Source of information and matching

The hospital records provided data on which the COVID-19-positive cases were matched to COVID-19 negative controls. These variables were age (± 5 years), sex (same sex) and date of testing (within the same month). Data were collected using a standardised structured questionnaire on REDCap via a telephone interview, maintaining the matched structure and recording further COVID-19 testing and vaccination information. In addition, the study recorded outcomes of COVID-19 disease as well as underlying comorbidities (diabetes, hypertension, heart disease, asthma, lung disease, HIV, kidney disease, cancer and liver disease).

### Data analysis

Data were exported into STATA software version 17 for Windows 11 for management, ensuring data cleanliness and consistency of responses. All normal continuous variables are presented as means (standard deviation [s.d.]) and medians (interquartile range [IQR]). All categorical variables (e.g. sex and age groups) are presented as frequencies and percentages.

We first describe the participant’s demographic and clinical-related characteristics by COVID-19 disease status (case vs. controls). We also present the (1) different vaccination status groups stratified by COVID-19 status; (2) the prevalence of comorbidities; (3) distribution of lifestyle characteristics (smoking status and alcohol intake); and (4) disease severity. Severe disease was classified as requiring oxygen while admitted, while moderate and mild were defined as hospitalised with no oxygen and home-based care, respectively.

Secondly, univariate conditional logistic regression models were implemented to determine the relationship between vaccination status and COVID-19 estimating the odds ratios (ORs) with the corresponding 95% confidence intervals (CI). Vaccine effectiveness was estimated as one minus the OR, for example, VE = (1 – OR)*100, computed from the primary analysis. Thirdly, further adjustments for *a priori* predictor confounding factors disease including smoking status, pregnancy (among females), sex and alcohol consumption were performed. Multicollinearity between predictor variables was assessed using the variance inflation factor (VIF < 5 indicating a good fit). Fourthly, to control for effect modification, stratified analyses for VE were conducted while adjusting for confounders. In a multiple conditional logistic regression model, effect modification was assessed by comparing the VE across strata using a likelihood ratio test for age group (different groups and group trend), presence of at least one chronic condition, vaccine brand and calendar time.

Fifthly, among COVID-19 positive participants, we described COVID-19 severity (mild, moderate and severe), management and disease outcome highlighting those who were hospitalised, home-based care or died. For COVID-19 severity, ordinal logistic regression models were used for risk estimation through ordered ORs with the corresponding 95% CI.

### Ethical considerations

Ethical clearance to conduct this study was obtained from the Medical Research Council of Zimbabwe (No. MRCZ/A/2987). Individual consent was sought from each participant, and written consent was obtained before each interview.

## Results

### Participant characteristics

The study enrolled 1000 participants on a 1:1 COVID-19 case-control ratio with an overall median age of 38 years (IQR: 30–50). More female participants were recruited (352 female pairs vs. 148 male pairs). Only 56 (5.6%) of the participants were healthcare workers (HCW) and responses from next of kin (NOK) were likely to be cases (32% vs. 13.8%) than controls (*p* < 0.001). The study also identified that almost three in four (74.4%) of the enrolled participants had a point-of-care COVID-19 test, and more cases tested using PCR test than controls (30.8% vs. 20.4%) (Table 1).

**TABLE 1:** Participant demographic characteristics.

Characteristics	Total (*N* = 1000)				COVID 19 (–) (*n* = 500)				COVID-19 (+) (*n* = 500)				*p*
	*n*	%	Median	IQR	*n*	%	Median	IQR	*n*	%	Median	IQR	
Age (years)	-	-	38	30–50	-	-	39	30–50.5	-	-	38	30–50	0.77
**Age (years)**	-	-	-	-	-	-	-	-	-	-	-	-	0.946
18–24	115	11.5	-	-	57	11.4	-	-	58	11.6	-	-	-
25–34	261	26.1	-	-	137	27.4	-	-	124	24.8	-	-	-
35–44	264	26.4	-	-	128	25.6	-	-	136	27.2	-	-	-
45–54	146	14.6	-	-	72	14.4	-	-	74	14.8	-	-	-
55–64	68	6.8	-	-	32	14.4	-	-	36	7.2	-	-	-
65–94	146	14.6	-	-	74	14.8	-	-	72	14.4	-	-	-
**Sex**													0.99
Female	704	70.4	-	-	352	70.4	-	-	352	70.4	-	-	-
Male	296	29.6	-	-	148	29.6	-	-	148	29.6	-	-	-
Health care worker	56	5.6	-	-	35	7.0	-	-	21	4.2	-	-	0.054
Response from NOK	229	22.9	-	-	69	13.8	-	-	160	32.0	-	-	< 0.001
**Test type**													< 0.001
PCR	256	25.6	-	-	102	20.4	-	-	154	30.8	-	-	-
Point-of-care	744	74.4	-	-	398	79.6	-	-	346	69.2	-	-	-
**Prior COVID-19 + test**	26	2.6	-	-	14	2.8	-	-	12	2.4	-	-	0.91
Yes	874	87.4	-	-	437	87.4	-	-	437	87.4	-	-	-
No	100	10.0	-	-	49	9.8	-	-	51	10.2	-	-	-
Do not know	-	-	-	-	-	-	-	-	-	-	-	-	-
**Current COVID-19**													< 0.001
vaccination status: fully	313	31.3	-	-	181	36.2	-	-	132	26.4	-	-	-
vaccinated/boosted: partially	67	6.7	-	-	15	3.0	-	-	52	10.4	-	-	-
Unvaccinated	620	62.0	-	-	304	60.8	-	-	316	63.2	-	-	-
**Number of COVID-19 vaccine doses**													< 0.001
1	68	17.7	-	-	15	7.6	-	-	53	28.2	-	-	-
2	211	54.8	-	-	112	56.9	-	-	99	52.7	-	-	-
3	101	26.2	-	-	69	35.0	-	-	32	17.0	-	-	-
Don’t know	5	1.3	-	-	1	0.5	-	-	4	2.1	-	-	-
**Brand of 1st dose**													0.16
J&J	2	0.5	-	-	1	0.5	-	-	1	0.5	-	-	-
Sinovac	206	54.2	-	-	96	49.0	-	-	110	59.8	-	-	-
Sinopharm	169	44.5	-	-	98	50.0	-	-	71	38.6	-	-	-
AstraZeneca	1	0.3	-	-	0	0.0	-	-	1	0.5	-	-	-
Sputnik	1	0.3	-	-	0	0.0	-	-	1	0.5	-	-	-
Covaxin	1	0.3	-	-	1	0.5	-	-	0	0.0	-	-	-
Presence of a chronic condition	322	32.2	-	-	146	29.2	-	-	176	35.2	-	-	0.042
Ever consumed alcohol	296	29.6	-	-	167	33.4	-	-	129	25.8	-	-	0.008
Ever smoke	82	8.2	-	-	43	8.6	-	-	39	7.8	-	-	0.645

NOK, next of kin; –, negative; +, positive; COVID-19, coronavirus disease 2019; J&J, Johnson & Johnson; PCR, polymerase chain reaction; IQR, interquartile range; COVID-19, coronavirus disease 2019.

Most of the participants (*n* = 874/1000) indicated that the documented result was their first time having a COVID-19 test. Data on vaccination status showed that 13.4% (*n* = 134) were fully vaccinated or had received a booster dose of the vaccine. In addition, one in four (*n* = 248; 24.8%) and three in five (*n* = 596; 59.6%) of the respondents were partially vaccinated or unvaccinated respectively. We also show that participants who were fully vaccinated and/or boosted (*n* = 134; 13.4%), were likely to be controls (*n* = 95; 19%) than cases (*n* = 39; 7.8%), partially vaccinated were likely cases (*n* = 146; 29.2%) than controls (*n* = 102; 20.4%) (Table 1).

Also, many of the participants had received two doses of a two-dose regimen (*n* = 211; 54.8%) and seconded by three doses (two-dose regimen plus a booster) (*n* = 101; 26.2%). In addition, the most common brands were Sinovac (*n* = 206; 54.2%) and Sinopharm (*n* = 169: 44.5%). Overall, a third of the participants (*n* = 322; 32.2%) reported having at least one of the chronic conditions.

The most common condition to be reported was HBP (18.4%) and this was followed by diabetes (9.5%) and HIV (6%). When asked about their smoking and alcohol intake status, most of the participants highlighted that they have never smoked (*n* = 913; 91.3%) or drank alcohol (*n* = 695; 69.5%). Comparatively more participants pointed currently taking alcohol (16.7%) versus smoking (2.5%), while 12.9% and 5.7% mentioned stopping alcohol and smoking respectively (Table 1).

### Vaccine effectiveness

Results from the conditional logistic regression model used to determine the association between vaccination status and COVID-19 disease status adjusted for age, date of testing and sex showed a statistically significant VE of 32.2% (95% CI: 8.9, 49.5) when compared against unvaccinated participants (Table 2). However, combining fully and partially vaccinated participants reduced the VE lacking statistical evidence (VE = 10.7%, 95% CI: –16.8, 31.7). On the other hand, comparing those fully vaccinated to a group of unvaccinated plus partially vaccinated showed a VE of 39.4% (95% CI: 19.1, 54.6) (Table 2). After stratified analysis, we have shown a lower VE for older participants and those with at least one chronic condition and no difference between Sinovac and Sinopharm vaccines (Table 2).

**TABLE 2:** Conditional logistic regression for the effect of vaccination on coronavirus disease 2019 status.

Analysis options	Vaccination status	OR	95% CI	*p*	VE	%
**Primary analysis**	Unvaccinated	Ref.	Ref.	-	-	-
	Fully vaccinated	0.68	0.50, 0.91	0.010	32.2	8.9, 49.5
	Partially vaccinated	3.33	1.79, 6.20	< 0.001	−232.9	−520, -78.8
**Secondary analysis**	Unvaccinated	Ref.	Ref.	-	-	-
	Fully + Partially vaccinated	0.89	0.68, 1.17	0.410	10.7	−16.8, 31.7
	Unvaccinated + Partially vaccinated	Ref.	Ref.	-	-	-
	Fully vaccinated	0.61	0.45, 0.81	< 0.001	39.4	19.1, 54.6
	Partially vaccinated	Ref.	Ref.	-	-	-
	Fully vaccinated	0.20	0.11, 0.39	< 0.001	79.6	61.1, 89.3
	Unvaccinated	0.30	0.16, 0.56	< 0.001	70.0	83.9, 44.1
**Age (years)**						
18–24 (ref)	Unvaccinated	Ref.	Ref.	-	-	-
25–34	Fully vaccinated	0.68	0.17, 2.68	0.581	32.1	−167.8, 82.8
34–44	Fully vaccinated	1.64	0.34, 7.89	0.535	−64.4	−689.3, 65.8
45–54	Fully vaccinated	2.59	17.55, 0.38	0.331	−155.9	−1655, 61.9
55–64	Fully vaccinated	-	-	-	-	-
65+	Fully vaccinated	-	-	-	-	-
**Age (trend)**	Unvaccinated	Ref.	Ref.	-	-	-
	Fully vaccinated	0.62	0.39, 0.98	0.041	38.3	2.0, 61.1
**Chronic condition:**						
No (ref)	Unvaccinated	Ref.	Ref.	-	-	-
Yes	Fully vaccinated	1.001	0.64, 1.56	0.998	−0.64	−55.7, 35.7
**Vaccine type**	Sinovac	Ref.	Ref.	0.555	−23.4	38.7, 148.5
	Sinopharm	1.23	0.61, 2.48	-	-	-

Ref., reference category; OR, odds ratio; CI, confidence interval; VE, vaccine effectiveness.

#### Coronavirus disease 2019 severity

**FIGURE 1:**
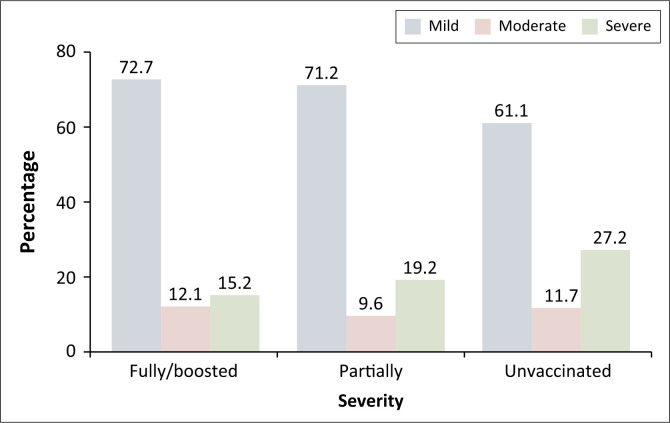
Disease severity among cases by vaccination status.

Of the 500 COVID-19 cases, most (*n* = 326; 65.2%) had mild disease, followed by severe (*n* = 116; 23.2%) and moderate (*n* = 58; 11.6%) disease. More specifically, more unvaccinated participants had severe disease (*n* = 86; 27.2%) versus partially vaccinated (*n* = 10; 19.2%) or fully vaccinated (*n* = 20; 15.2%) (Figure 1).

### Coronavirus disease 2019 management (home-based care or hospitalised) and outcome (recovered or died)

Of the 500 cases enrolled in the study, 326 (65.2%) of the participants were managed at home while the remainder (*n* = 174) were hospitalised (Table 3). Participants who were hospitalised were older [median: 46; IQR: 35–65] than those who were home-based cared [median: 36.5; IQR: 30–46]. More participants with a chronic condition were hospitalised (49.4%) as compared to being provided care at home (27.6%). More so, of the 174 participants who were hospitalised, the majority (*n* = 95; 54.6%) were admitted to hospital for less than a week. In addition, only seven of the 174 participants (4.0%) were in hospital for more than 2 weeks. In addition, two-thirds of the participants required oxygen during admission and 40% of those hospitalised were admitted in the intensive care unit. Notably, 11.6% (*n* = 58) of the participants died among cases and there was no control mortality reported (Table 3).

**TABLE 3:** Coronavirus disease 2019 management and outcome among cases.

Variables	Total (*n* = 500)				Home-based cared (*n* = 326)				Hospitalised (*n* = 174)				*p*
	*n*	%	Median	IQR	*n*	%	Median	IQR	*n*	%	Median	IQR	
Age (years)	-	-	38	30–50	-	-	36.5	30–46	-	-	46	35–65	< 0.001
Sex (female)	352	70.4	-	-	235	72.1	-	-	117	117 (67.2)	-	-	0.260
Presence of a chronic condition	176	35.2	-	-	90	27.6	-	-	86	86 (49.4)	-	-	< 0.001
**Length of stay in hospital (weeks)**													
< 1	95	19.0	-	-	-	-	-	-	95	54.6	-	-	-
1–2	72	14.4	-	-	-	-	-	-	72	41.4	-	-	-
> 2	7	1.4	-	-	-	-	-	-	7	4.0	-	-	-
Required oxygen during admission	116	23.2	-	-	-	-	-	-	116	66.7	-	-	-
Admitted in the intensive care unit	71	14.2	-	-	-	-	-	-	71	40.8	-	-	-
COVID-19 outcome (death)	58	11.6	-	-	13	4.0	-	-	45	25.9	-	-	< 0.001

IQR, interquartile range; COVID-19, coronavirus disease 2019.

In the multivariable analysis, we have shown that after co-adjusting the factors in the bivariable models, the effects of vaccination, being a HCW and alcohol intake were attenuated (Table 4). Regardless, it should be noted that the oldest participants (65+ years) were three times more likely to be severely diseased when compared to the youngest age group (18–24 years). More so, after mutually adjusting for confounders and potential effect modification, presence of a chronic condition was twice likely associated with severe COVID-19 disease (aOR = 1.86, 95% CI: 1.20, 2.89) than combined moderate and mild cases. Similarly, participants who reported either having stopped (aOR = 2.55, 95% CI: 1.03, 6.29) or currently smoking (aOR = 2.30, 95% CI: 0.71, 7.44) were at least twice more likely to report a severe case (Table 4).

**TABLE 4:** Ordinal logistic regression for factors associated with COVID 19 severity (*n* = 500).

Variable	Univariable analysis		Multivariable analysis		*p* ^AOR^
	OR	95% CI	aOR	95% CI	
**Vaccination status**					
Unvaccinated	Ref	Ref	Ref	Ref	-
Fully vaccinated	0.56	0.36, 0.87	0.68	0.41, 1.11	0.124
Partially vaccinated	0.63	0.34, 1.19	0.92	0.45, 1.86	0.808
**Age (years)**					
18–24	Ref	Ref	Ref	Ref	-
25–34	0.40	0.20, 0.81	0.46	0.21, 1.01	0.053
35–44	1.07	0.57, 2.01	1.01	0.50, 2.02	0.984
45–54	1.32	0.65, 2.66	1.07	0.49, 2.36	0.859
55–64	2.14	0.94, 1.90	1.62	0.64, 4.05	0.306
65–94	3.69	1.85, 7.35	3.58	1.62, 7.91	0.002
Sex (male)	1.32	0.89, 1.95	0.79	0.48, 1.31	0.363
Presence of at least one chronic condition	2.65	1.82, 3.85	1.86	1.20, 2.89	0.005
HCW	0.30	0.09, 1.04	0.36	0.09, 1.49	0.159
Ever tested (+) for COVID 19 before	0.80	0.21, 2.95	1.80	0.41, 7.96	< 0.001
**Smoking status**					
Never smoked	Ref	Ref	Ref	Ref	-
Stopped	4.11	1.94, 8.68	2.55	1.03, 6.29	0.043
Currently smoking	1.66	0.62, 4.40	2.30	0.71, 7.44	0.163
**Alcohol intake**					
Never took alcohol	Ref	Ref	-	-	-
Stopped	2.55	1.50, 4.31	1.19	0.61, 2.30	0.612
Current drinker	1.31	0.78, 2.23	1.36	0.67, 2.78	0.392

HCW, health care workers; OR, odds ratio; aOR, adjusted odds ratio; CI, confidence interval; Ref, reference category.

## Discussion

The study identified a primary VE of 32% among fully vaccinated individuals when compared against those unvaccinated. The VE reduced among the elderly, and was negative among participants with a chronic condition like hypertension and diabetes reduced it. In addition, most COVID-19 cases were managed at home, and these were likely to have received at least one COVID-19 dose (partially vaccinated). Likewise, age, being a nurse and the presence of a chronic condition were associated with COVID-19 hospitalisation. However, COVID-19-related mortality was low though associated with higher age, sex, presence of a comorbid condition and testing COVID-19 positive at a prior event.

This study indicated a VE of 32% among fully vaccinated participants (Sinovac and Sinopharm) and this is lower than what has been reported in similar settings such as in Jordan where patients admitted to Prince Hamza Hospital showed a VE of 67%.^17^ Nonetheless, in a different study in Latin America, CoronaVac an equivalent to Sinovac was identified to have quite a moderate VE of 53%.^18^ Comparatively, other studies have also shown that participants who were recipients of other vaccines (Pfizer-BioNTech/Comirnaty) were less likely to get infected than those on Sinovac-CoronaVac and Sinopharm.^19^ Recent studies have highlighted that the emergence of COVID-19 variants like Delta and Omicron lowered VE, particularly impacting inactivated vaccines such as Sinovac and Sinopharm. More so, the presence of chronic conditions like diabetes was found to diminish COVID-19 VE, a trend observed in other studies as well.^20^ Our retrospective findings on VE are comparable to the clinical results elsewhere including countries that were not necessarily offering the vaccines to their local population. Taking into consideration the fact that the Sinopharm clinical trials yielded a 79% efficacy according to WHO,^21^ our findings would translate to acceptable effectiveness.

Most COVID-19 patients were managed at home, and this could be attributed to a myriad of factors including but not limited to lack of health service access and having received at least one COVID-19 dose. Similarly, in a low-resource setting in Pakistan, 80% – 85% of COVID-19 patients were reported to have mild disease and were home treated.^22^ Likewise, data from the Vizient clinical database in 2020 (United States) showed that more than two-thirds of COVID-19 patients were managed in an outpatient setting.^23^ Partly this can be explained by the fact that most COVID-19 patients experienced mild to moderate symptoms as observed in this study because of a robust immune response, low viral load infection, vaccination or prior exposure.^24^ Consistent to our findings, a retrospective cross-sectional analysis of an open-access database on African COVID-19 cases also identified that older age and presence of chronic disease were associated with worse COVID-19 outcomes.^25^ Notably, in older age one is more likely have a weaker immune system thus becoming more susceptible to the infections like acute respiratory syndrome and, subsequently, death.

More importantly, the COVID-19-related mortality rate of 11.6% identified in this study was higher than what has been reported in other parts of sub-Saharan Africa (2.4%).^26^ As reported by Bradshaw (2022),^27^ it is possible that many African settings have underestimated COVID-19-related mortality because of poor surveillance and health reporting systems. Nonetheless, it is also important to highlight that our study could have over-estimated the COVID-19-related deaths since sampling was done from hospitals; cases presenting at hospitals were likely severe; hence the denominator for COVID-19-related mortality for calculating mortality rate was inappropriate. Interestingly, the local Ministry of Health through their weekly situation report on COVID-19 (02 December 2023) has reported a COVID-19 case fatality rate of 2.1% which is comparable to global estimates. However, similar findings have also shown that sex, age and presence of a comorbid condition are important predictors of COVID-19 mortality.^28,29^ Likewise, elderly populations may be disproportionately affected by COVID-19 owing to fragility because of ageing and physiological changes, weaker immunity compared with younger people and the increasing frequency of non-communicable comorbidities associated with age.

Study limitations include the retrospective design which is prone to recall bias though data from participants were cross-referenced with hospital records to improve their quality. Hospital records were also prone to missingness making follow-up of participants difficult. Assessment of VE in older participants was limited as indicated by uncertain estimates, characterised by wider CI because of a smaller sample size within that category. In addition, the study lacked data on time of infection and overall availability of treatment among participants that could have improved understanding of COVID-19 disease outcomes.

## Conclusion

We have shown a VE in this setting of 32%. Our findings have also highlighted the association between age, chronic comorbidities and COVID-19 severity such that understanding these complexities is essential for personalised healthcare approaches to improve health outcomes. More so, we have identified that just about half of the participants were vaccinated, emphasising the importance of encouraging more people to be vaccinated and completing the vaccination series.

## Recommendations

Given the observed effectiveness variation in different demographics, the Ministry of Health (MOH) should consider tailoring vaccination campaigns to focus on high-risk groups like the elderly and individuals with chronic conditions. In addition, the MOH incorporates targeted messaging on the importance of completing the vaccination series to mitigate breakthrough infections as well as finding ways to mitigate misinformation. In addition, we also recommend comprehensive strategies to manage chronic conditions like hypertension and diabetes, which were found to reduce VE. This may involve specialised vaccination protocols, ongoing monitoring and tailored healthcare approaches for individuals with such conditions. On the other hand, investing in ongoing surveillance and research efforts to monitor breakthrough infections among partially vaccinated individuals. These data are crucial for understanding the dynamics of VE over time and against emerging variants.
